# A universal indicator of critical state transitions in noisy complex networked systems

**DOI:** 10.1038/srep42857

**Published:** 2017-02-23

**Authors:** Junhao Liang, Yanqing Hu, Guanrong Chen, Tianshou Zhou

**Affiliations:** 1 School of Mathematics, Sun Yat-Sen University, Guangzhou 510275, P.R. China; 2 School of Data and Computer Sciences, Sun Yat-Sen University, Guangzhou 510275, P.R. China; 3 Department of Electronic Engineering, City University of Hong Kong, Kowloon, Hong Kong SAR, P.R. China; 4 Key Laboratory of Computational Mathematics, Guangdong Province, Guangzhou 510275, P.R. China

## Abstract

Critical transition, a phenomenon that a system shifts suddenly from one state to another, occurs in many real-world complex networks. We propose an analytical framework for exactly predicting the critical transition in a complex networked system subjected to noise effects. Our prediction is based on the characteristic return time of a simple one-dimensional system derived from the original higher-dimensional system. This characteristic time, which can be easily calculated using network data, allows us to systematically separate the respective roles of dynamics, noise and topology of the underlying networked system. We find that the noise can either prevent or enhance critical transitions, playing a key role in compensating the network structural defect which suffers from either internal failures or environmental changes, or both. Our analysis of realistic or artificial examples reveals that the characteristic return time is an effective indicator for forecasting the sudden deterioration of complex networks.

Critical transitions occur in many complex networked network systems ranging from ecosystems^1^, climate systems^2^, global finance^3^, to complex diseases^4^,^5^,^6^. Once this transition takes place, the underlying system goes across a critical threshold (or a tipping point) and then shifts to a new stable state dramatically different from the original stable state. Near the tipping point, there are early-warning signs, such as slower recovery from perturbations^7^,^8^, higher autocorrelation^9^,^10^ and larger variance^11^. Such a so-called “critical slowing down” is an interesting dynamical phenomenon occurring in the vicinity of a tipping point where the system becomes increasingly slow when recovering from a slightly perturbed state back to its equilibrium state. Methods for detecting critical transitions based on the theory of critical slowing down have been applied to many scientific fields such as chemistry^12^, physics^13^,^14^, engineering^15^,^16^, climatology^17^, ecology^8^,^18^,^19^,^20^, microbiology^21^, physiology^22^, and sociology^23^.

Although these methods can well forecast when critical state transitions occur in some deterministic systems, but the critical slowing down phenomenon is not a universal mechanism. For instance, when a stochastic system is close to its tipping point, a phenomenon called “flickering”^24^,^25^ may occur, that is, the system may switch between several potential states due to the effect of stochastic fluctuations, leading to multimodality or even vibration. This is actually another kind of early-warning signal that in general cannot be predicted by the occurrence of critical slowing down. Another issue is that noise may modify or even completely change the behavior of a deterministic system, e.g., noise-induced stochastic switching^26^, noise-induced oscillation^27^, and noise-induced stochastic synchronization^28^. Thus, a natural question is how one can predict critical state transition in a noisy dynamical system.

We will address this issue by considering a homogeneous complex network (where nodes are both similarly and highly connective) subjected to noise perturbations. Our consideration is based on two reasons: (1) stochastic fluctuations (or noise) exist extensively in many real-world complex networks, e.g., biochemical reaction networks are inherently noisy due to low copy numbers of reactive species^29^,^30^,^31^; (2) homogeneous networks tend to evolve through critical transitions in response to external or environmental changes, whereas heterogeneous networks (where nodes are dissimilar but modular) tend to change gradually, implying that no critical transition happens^25^,^32^,^33^,^34^. For a noisy homogeneous complex network, we will develop a theoretical framework for forecasting how a critical state transition can occur and how noise affects this transition. This analytical framework is inspired by a recent work of Gao *et al*.^35^, which studied the resilience patterns in deterministic complex networked systems. We first show how the dynamical behavior of a multi-dimensional noisy complex networked system can be captured by a one-dimensional (1-D) indicative equation. Then, using the linear noise approximation^36^, combined with bifurcation theory^20^,^37^, we show how a critical-slowing-down signal arises when the higher-dimensional system approaches its critical transition. In particular, by the effective stability approximation^38^, we derive an analytical expression for the noise-dependent characteristic return time defined as the negative inverse of the mean characteristic value of the 1-D indicative system. This characteristic time can well capture early-warning signals of the original large-scale system since it tends to infinity when the system approaches to a tipping point, and hence it can be taken as an effective indicator of critical transitions. In fact, our numerical results have verified that this theoretical characteristic return time of the 1-D system is in good accordance with the actual characteristic return time of the original networked system (see the Results section). More importantly, this indicator allows us to systematically separate the respective roles of the dynamics, noise and topology of a complex network in controlling its sudden deterioration.

Numerical analysis of examples shows that for complex networks, random failures (or attacks) drive critical transitions in an elusory way, but their dynamic features can always be captured by the corresponding 1-D indicative system. In particular, a dramatic change in the characteristic return time in the 1-D system well anticipates the upcoming critical transition in the underlying complex networked system. Moreover, the numerical results show that a higher intensity of noise leads to a smaller possibility for the occurrence of critical transitions, implying that noise has a potential to compensate the structural defect of a complex network suffered from either internal failures or environmental changes. It should be emphasized that although these results are obtained by analyzing specific examples, they are qualitatively invariant for other similar complex networks, therefore practically useful.

## Method

A noisy homogenous complex networked system can be described by the following coupled stochastic differential equations^39^,^40^:


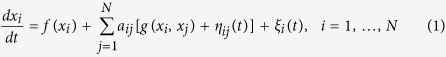


where *x*_*i*_ describing the dynamics of node *i* may represent a certain quantity of a species, the expressional level of a gene, etc.; the element *a*_*ij*_ in the network adjacency matrix 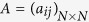 represents the weight of the directed edge from node *j* to node *i*; *f(x*_*i*_) is the primitive deterministic dynamics of node *i*; *g(x*_*i*_, *x*_*j*_) represents the influence of node *j* on node *i* (hence, *a*_*ij*_*g(x*_*i*_, *x*_*j*_) represents the effective influence); *ξ*_*i*_(*t*) models fluctuations in the external environment (called external noise) and *η*_*ij*_(*t*) models the uncertain of the influence of node *j* on node *i* (called internal noise).

Note that Eq. (1) is a higher-dimensional system with the dimension equal to the number of the network nodes, so it is in general difficult to analyze and compute. However, we will show that the global (mean) dynamics of this system can be well captured by a 1-D system for the mean of {*x*_*i*_} over the network if the functions *f*(·) and *g*(·, ·) satisfy some constrained conditions (see Supplementary Information A.2 for details). Specifically, if we define 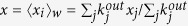 with 
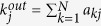
 representing the out-degree of node *j*, then the dynamics of *x* will be governed by the following 1-D equation, called the indicative system (see Supplementary Information A.2 for derivation):


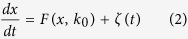


where 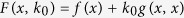 and 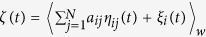. Properties of the noise *ζ(t*) depend on the original noisy sources {*ξ*_*i*_} and {*η*_*ij*_}. In Eq. (2), the parameter 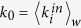 represents the weighted average of the in-degree 
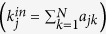
 over all the nodes. It can be observed that Eq. (2) well separates the roles of the original large-scale system dynamics, network topology and noise, since (i) the function *F(x, k*_0_) is a linear combination of two functions, *f*(·) and *g*(·, ·); (ii) the parameter *k*_0_ represents some topological characteristics of the original complex network; (iii) the original diverse noisy sources are currently captured by a single noise, *ζ(t*). It is worth pointing out that when Eq. (2) is derived from Eq. (1), there is no limitation on noisy sources, e.g., they may be correlated to each another.

If the intensity of noise *ζ(t*) is small enough, then using the linear noise approximation^36^ and the effective stability approximation^38^, we can derive an explicit expression for the effective eigenvalue of Eq. (2) (denoted by 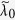) at a stable equilibrium (denoted by *x*_*s*_). In fact, we have (see Supplementary Information A.1 for derivation)


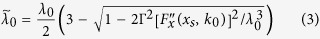


where *λ*_0_ is the characteristic value of the unperturbed system 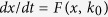, and 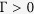 is the intensity of noise *ζ(t*). From Eq. (3), it can be observed that for a small negative *λ*_0_ (implying that the original unperturbed system is stable), the noise may have a positive contribution to the effective eigenvalue. Moreover, some intensities of the noise can let 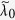 approach zero (implying that the original stability is losing) and then become positive (implying that the system has stepped over the critical state).

Since the sign of a characteristic value close to zero is difficult to detect in practical cases, we define


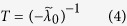


This quantity, called an indicator, actually represents the characteristic return time of the 1-D indicative system (2) since the mean behavior of *x(t*) is governed by 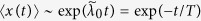 (see Supplementary Information A.1 for derivation). This indicator allows us to separate the respective roles of the system dynamics, network topology and noise, as discussed above. In particular, it can better forecast when critical transition occurs than 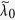. In fact, *T* will diverge to positive infinity when the characteristic value 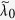 approaches zero from the negative direction, and negative infinity when 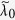 approaches zero from the positive direction. Here a characteristic value approaching zero implies that the system approaches a critical state. In addition, the indicator can also describe the resilience ability of the dynamical system. Generally, near a critical state, the system tends to have less resilience ability, as indicated by the critical slowing down. An abrupt increase of *T* implies that the system has a slower recovery rate from perturbations, and a negative *T* implies that the system has lost its recovery ability.

We should point out that there are two possible kinds of fold bifurcations for the deterministic system corresponding to Eq. (2), referring to Fig. S3 in the Supplementary Information. Since the links between nodes represent the interactive effects in a complex network, the topological characteristic parameter *k*_0_ represents the strength of such effect. If a greater *k*_0_ (representing a stronger interactive effect) leads to a larger *x*_*s*_, implying that the interactive effect is enhanced, then the corresponding bifurcation is mutualistic. On the contrary, if the interactive effect is repressed, then the corresponding bifurcation is competitive. Note that the type of the interactive effect is determined by function *g(x, y*), that is, 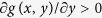 corresponds to the mutualistic effect whereas 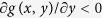 to the competitive effect. Our theory discussed above holds for both kinds of interactive effects.

To better understand the above theory and show relevant results more clearly, let us consider a representative example with mutualistic interaction, where the functions in Eq. (1) take the following forms:


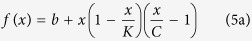



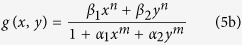


The function *f(x*) which takes the form of the Logistic growth with Allee effects^41^,^42^ describes a general growth mode, whereas the interactive function *g(x, y*) which is a generalized Hill function^43^ describes the underlying process.

In the following, for the analysis and simulation convenience but without loss of generality, we assume that *ξ*_*i*_(*t*) is the Gaussian white noise, satisfying the so-called *δ*–correlation: 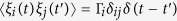, where *δ*_*ij*_ is the Kronecker delta and *δ*(·) is the common delta function, and that *η*_*ij*_(*t*) is also the Gaussian white noise, satisfying the correlation 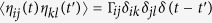. In addition, we assume that the noise intensities 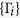, 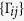 are all constants, and that internal and external noisy sources are mutually independent, i.e., 
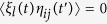
 for all *i, j, l* and all *t, t*′.

The 1-D indicative system corresponding to Eq. (1) currently becomes


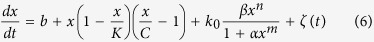


where *α* = *α*_1_ + *α*_2_, *β* = *β*_1_ + *β*_2_, 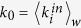. Furthermore, we can derive an analytical expression for the characteristic return time *T*. For example, for *n* = 2 and *m* = 1, we have


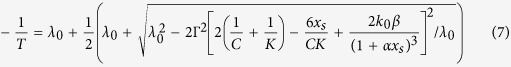


where 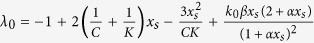, *x*_*s*_ is a positive root of the algebraic equation 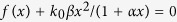, and 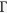, the intensity of the *ζ(t*) noise, is given by 
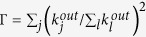

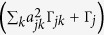
.

**Figure 1:**
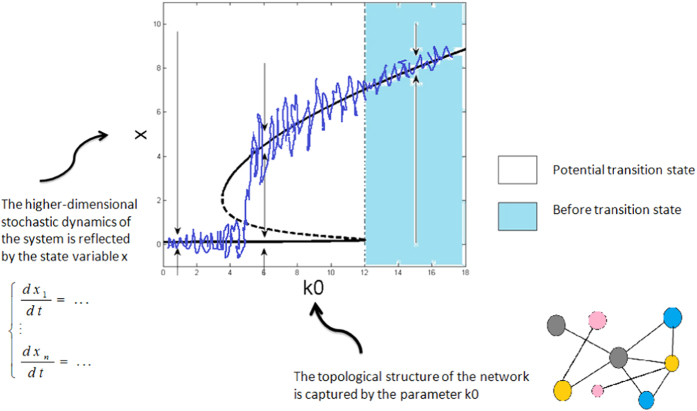
Schematic diagram for the dynamics of the system corresponding to Eq. (6). The solid line and dashed line represent stable and unstable equilibria respectively. At the before-transition state, the system has its position around a unique stable equilibrium (with a large magnitude), possessing strong resilience against perturbations. With the decrease of *k*_0_, the deterministic system goes through a fold bifurcation and three equilibria (two stable and one unstable) may coexist, implying a potential critical transition. However, noise can tune the position of the bifurcation point.

Note that if *k*_0_ is used as a bifurcation parameter, then the system (6) can go through a deterministic fold catastrophic bifurcation^24^,^37^ (see Supplementary Information B for analysis). As a result, at the before-transition state, the system has its position around a unique stable equilibrium with a large magnitude, possessing strong resilience. As *k*_0_ decreases, a new stable equilibrium (with a small magnitude) may appear, implying that the critical transition may happen. However, the existing noise can change the position of the bifurcation point, referring to Fig. 1.

To show the effectiveness of our prediction theory, we numerically analyze three realistic complex networks in ecology^44^ and molecular biology^45^. Analysis of six additional artificial complex networks is reported in Supplementary Information D.1. The descriptions (including construction methods and weight assignments) of all networks data used in analysis are put in Supplementary Information C.1. We emphasize that our prediction theory (namely, the indicator *T* can well forecast when critical transition occurs) is verified by analyzing a few specific complex networks, but the qualitative results obtained hold also for other similar complex networks.

## Results

### Random failures drive critical transitions in an elusory way

By random failure or attack we mean that nodes of a fraction *ρ* are deleted from a complex network randomly or that edge weights of a proportion *ρ* are decreased randomly (see Supplementary Information C.2). Each of these two ways can describe the loss of a functional network and therefore can be used to model the internal failures or external environmental deteriorations of the network. Here, we analyze the effect of random attacks on critical transitions in a complex network, testing whether there is a steady state with a small magnitude if the original system is at a steady state with a large magnitude (see Supplementary Information C.2 for explanation). The results are show in Fig. 1. The effect of random failures can be similarly analyzed and the obtained results are similar too.

**Figure 2:**
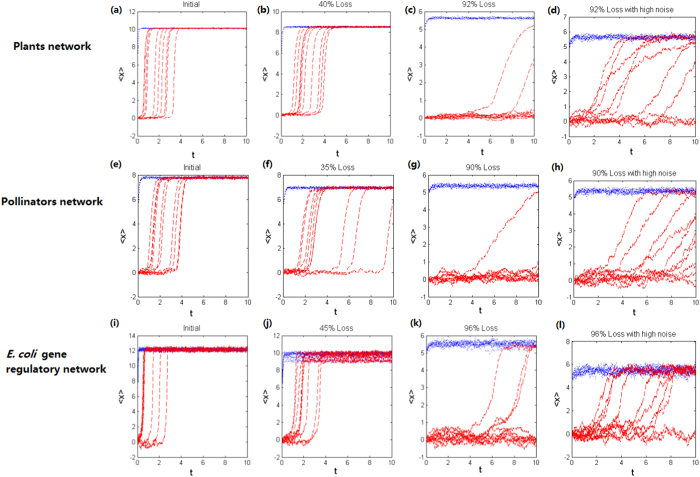
Critical transitions in complex networks after they are failed or attacked. Greater fraction of network loss drives the system to be closer to the critical transition. However, the randomness of perturbations or the noise can cause diverse dynamical behaviors of the system, hindering prediction of critical transition. The red/blue curves correspond to the evolutional processes with low/high initial state values. (**a**–**d**) Plants mutualistic network; (**e**–**h**) Pollinators mutualistic network; (**i**–**l**) Gene regulatory network of E. coli. The parameter values are set as: (**a**–**c**) and (**i**–**k**) 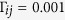, 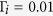; (**d**,**h**,**l**) 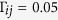, 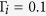; (**e**–**g**) 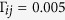, 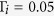. In all cases, *b* = 0.1, *C* = 1, *K* = 5, *n* = 2, *m* = 1, *n* = 2, *m* = 1, *α*_1_ = *β*_1_ = 0.01, *α*_2_ = *β*_2_ = 0.19. Each graph includes 10 random realizations.

Numerical simulations (Fig. 2) show that when suffered from a small loss (i.e., a small *ρ*), the complex networked system has its position around a unique stable state with a large magnitude and its resilience is maintained. With the increase of loss, the system becomes closer to a critical state and the resilience ability becomes weaker. After the network is weakened to a certain degree, the critical transition occurs and another stable state with a small magnitude appears, implying that the system has lost its resilience. If the network is severely deteriorated, then the whole system will collapse. Moreover, this process is irregular and unpredictable due to either the randomness of the failing realization or the effect of noise, or both.

From Fig. 2, one can observe another interesting phenomenon. Although there is a stable state with a small magnitude after the network is weakened to some degree, the mean 〈*x*〉 may still return to the original stable state with a large magnitude. This is because the mean 〈*x*〉 can be impacted by stochastic fluctuations and the superposition of these fluctuations can drive the system back to the attractive basin of the stable equilibrium with a large magnitude. Thus, although the network loses its topological structure when failed, the existing noise may compensate this loss. However, the recovery rate is much lower than that in the normal case where the network’s topological structure is not deteriorated.

### The characteristic return time is a good indicator of critical transitions

**Figure 3:**
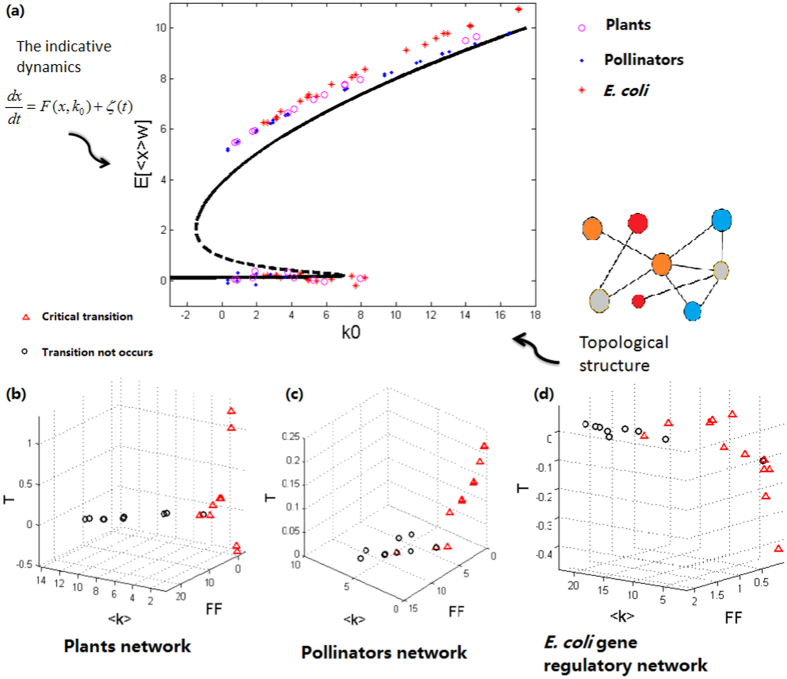
A universal indicator of critical state transitions. (**a**) The 1-D indicative equation can well capture the stochastic behavior of a higher-dimensional complex networked system. The solid curve corresponds to the theoretical weighted mean value predicted by the 1-D indicative equation (6) whereas the dots to the actual weighted mean values obtained by running different networked systems. (**b**–**d**) The characteristic return time *T* can well forecast for the occurrence of critical state transitions, that is, when the system approaches a critical state, *T* tends to a sudden decrease and even may become negative. Here, 〈*k*〉 represents the mean degree of the network and 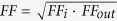 is the Fano Factor (see Supplementary Information A.2 for the definitions of the symbols). The symbolic points correspond to the cases of different loss fraction values of *ρ* (see Supplementary Information C.2 for more details), which are set as 0.1, 0.2, 0.3, 0.4, 0.6, 0.7, 0.8, 0.85, 0.9, 0.95 and 2 random failures realizations are simulated for each *ρ*. These different loss fraction values lead to different 〈*k*〉 and *FF*. Parameter values are set as *b* = 0.1, *C* = 1, *K* = 5, *n* = 2, *m* = 1, *α*_1_ = *β*_1_ = 0.01, *α*_2_ = *β*_2_ = 0.19 for all networks; 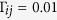, 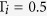 for the Plants and Pollinators networks, and 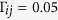, 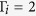 for the E. coli gene regulatory network.

When a fraction *ρ* of the nodes is randomly removed (or the weight of the links is randomly reduced by a mean percentage *ρ*), the network changes its topology in a random way, leading to changes in the value of the topological characteristic parameter *k*_0_. In order to further test our theory, we let *ρ* be changed from 0% to 100% in simulation, thus getting different values of *k*_0_. We find that the simulating results on the relationship between the parameter *k*_0_ and the expectation *E*[〈*x*〉_*w*_] (see Supplementary Information C.2 for explanation) are in good accordance with those predicted through the indicative system (6), referring to Fig. 3. This verifies that our theory is indeed effective.

In each realization, one can easily calculate the characteristic return time *T* according to Eq. (7). The results in Fig. 3 show that, in the case where critical transitions occur, *T* has a sudden increase, which may even become negative, also in accordance with the prediction of our theory (see Supplementary Information A). Although the change of *T* seems irregular and even unpredictable when the network responds to random failures, the notable change mode confirms that *T* is a good indicator of critical state transitions. In addition, the results shown in Fig. 3 also confirm our prediction on the relationship between the characteristic return time and the network topology (Supplementary Information Table S1).

**Figure 4:**
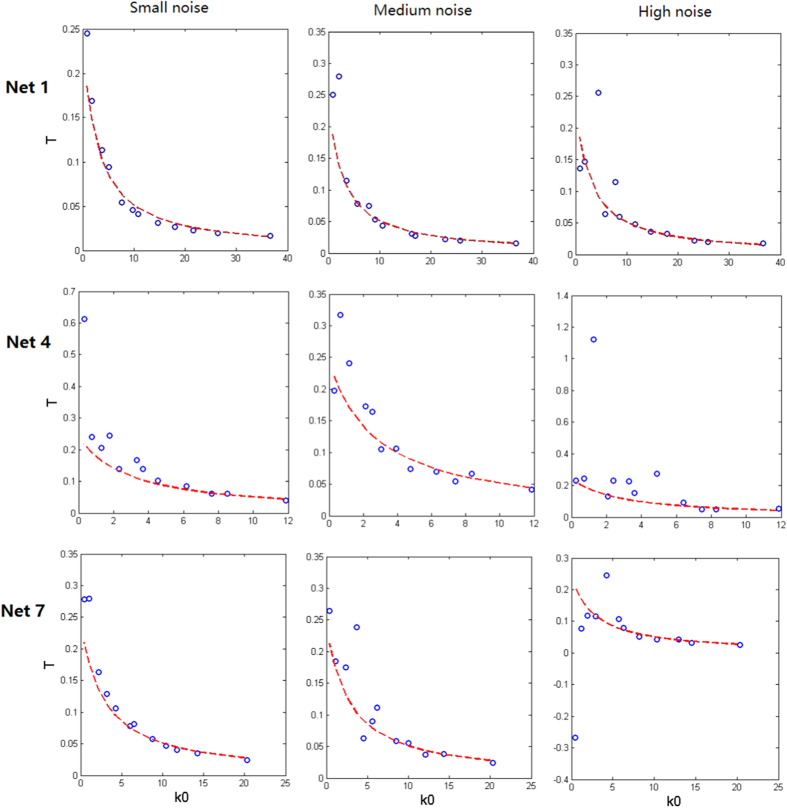
Comparison between the theoretical and actual characteristic return time *T*, where dashed (red) curves represent the theoretical result given by Eq. (7) whereas empty circle (blue) represent the actual one of the networked system. Parameter values are set as 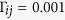, 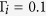 in small noise cases, 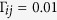, 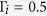 in medium noise cases, and 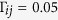, 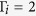 in high noise cases. The loss fraction *ρ* is set as 0, 0.3, 0.4, 0.5, 0.6, 0.7, 0.75, 0.8, 0.85, 0.9, 0.95, 0.98 first from left to right for every row and then from top to bottom, respectively. Other settings are the same as Figs 2 and 3.

In order to show the power of our method, we compare the theoretical characteristic return time given by Eq. (4) with the actual one obtained directly by the original networked system. Specifically, for 3 realistic networks (Net1, Net4 and Net7), we calculate the theoretical characteristic return times according to Eq. (7) and the actual return times obtained directly by simulating the networked systems. The numerical results are shown in Fig. 4, where the row diagrams from top to bottom correspond to Net1, Net4 and Net7 respectively, and the column diagrams from left to right correspond to small noise, medium noise and high noise respectively. From this figure, it can be seen that the theoretical characteristic return time (dashed lines) agrees well with the actual one (empty circles). Thus, the theoretical indicator derived in this paper indeed can well forecast critical transitions in realistic networked systems.

### Noise can compensate the structural defects of complex networks

**Figure 5:**
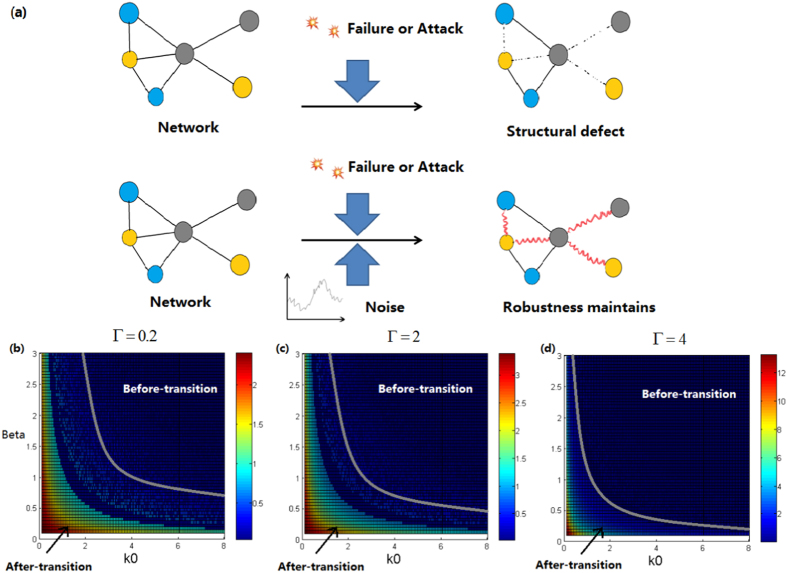
Noise can compensate the structural defect of a failing network. (**a**) Schematic diagram for the compensative effect of noise; (**b**–**d**) Parametric phase graph of the system, where the grey curves represent the phase boundaries. The Right-Up/Left-Down regions bounded by the curves are the stability ranges corresponding to before/after transition state. The value of *T* (which has been magnified 10 times) is showed by the color. The noise intensities of (**b**–**d**) are Γ = 0.2, 2, 4 respectively and some parameter values are set as *b* = 0.1, *C* = 1, *K* = 5, *n* = 2, and *m* = 1, *α* = 0.2.

Critical transition can be characterized by the stability phase since the system is monostable at the before-transition state and bistable at the after-transition state. Since the 1-D indicative system (6) can well capture the essential dynamics of the original large-scale noisy complex networked system, here we further investigate how the noise affects the critical transition in such a 1-D system. For this purpose, in our simulations we change the values of the topological characteristic parameter *k*_0_ and the effective parameter *β* which quantifies the strength of the interactive process among network nodes. The numerical results are shown in Fig. 5(b–d), where the effects of noise are reflected by the curves that represent the phase boundaries of the indicative system. Figure 5a is a schematic diagram showing the network structural defect, as well as how noise can compensate this defect.

It can be observed from Fig. 5(b,c), and d that a higher intensity of noise tends to creating a smaller parametric range for the after-transition state, implying that noise can delay critical transitions (this qualitative result can also be seen from the analytical expression for the negative inverse of the characteristic return time, i.e., Eq. (7)). In such a way, noise plays a role of compensating the structural defect of a failing network, where the defect results from either internal failures or environmental deteriorates, or both.

Again, we stress that the results in Fig. 5(b–d), although obtained by analyzing a specific example, are universal, independent of the network structures and dynamics of the deterministic system (i.e., functions *f(x*) and *g(x, y*)).

By summarizing the above analysis, we conclude that random failures or attacks can result in resilience loss of noisy complex networked systems and make them shift to critical states. Although this process seems irregular and unpredictable due to the stochastic effect of noise or due to random realizations, the indicator *T* can still well forecast the critical transitions. In addition, noise has a potential to compensate the structural defect of a failing network.

## Discussion

Many complex networked systems are often perturbed by noise, and can shift from one stable state to another different, often undesirable state (i.e., critical transition), giving rise to a question of how one can reliably predict the occurrence of such critical state transitions. In this paper, we have first shown how the dynamical behavior of a higher-dimensional noisy complex networked system can be captured by a 1-D indicative equation (i.e. Eq. (2)). Then, we have derived an analytical expression for the characteristic return time (as an indicator) for the 1-D system, which incorporates the effect of noise (see Eq. (4) with Eq. (3)). This noise-dependent indicator can be easily calculated through the given network data, which allows us to systematically separate the roles of the higher-dimensional system dynamics, noise and network topology in controlling critical transitions. As such, it provides a good measure to detect or cope with critical state transitions in complex networked systems subjected to noise perturbations.

Traditionally, the intrinsic behavior of the components (or the nodes) in a complex network was viewed invariable to perturbations. Perturbations only affect the structure of the network, determining who interacts with whom and how strong the interactions are. We have shown that noise, a kind of perturbation, can tune a networked system’s tipping point and play a role of compensating the structural defect of a failing network due to either internal failures or environmental changes or both. Thus, we conclude that noise is an unneglectable factor impacting the behavior of complex networked systems and therefore needs to be handled carefully.

Our theory on detecting the critical transitions in homogeneous networks offers a new way to enhance or prevent such transitions. First, the topological characteristic parameter *k*_0_ in the 1-D indicative system preserves all the topological properties of the underlying network. Second, the function *F(x, k*_0_) in Eq. (2) incorporates the dynamics of all individual components in the network. Third, Table S1 in the Supplementary Information lists all the key topological properties of networks, including those that can decrease the characteristic return time 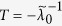 or can prevent the occurrence of critical transitions. All these can guide decision making and repair of the failing networks. In particular, the indicator *T* suggests a potential intervention strategy to avoid the loss of a desired state, and provides a design principle for optimal control systems that can well cope with perturbations.

In our theoretical framework, we used the linear noise approximation^36^ and the effective stability approximation^38^ to derive the analytical results, including the analytical expression for the characteristic return time. Although these results extend previous works, these approximations are valid only for small fluctuations. One unsolved problem is how one can predict critical state transitions in a complex network with larger-magnitude noise since the large deviation theory is invalid in this case.

Finally, our analytical framework provides a feasible and effective way to detect the critical transitions in complex networked system, but some issues remain to be addressed. For example, to what extent does our theory lose its prediction ability when a complex network is not homogenous? Can it still work for more general forms of node dynamics? Indeed, in a generic (heterogenous) system of complex network, one would expect that the characteristic return time studied in this paper to be non-existent, and even if existing, it would depend on which direction in a high-dimensional, asymmetric potential landscape that the system is switching from one stable state to another or vice versa due to the effect of stochastic fluctuations. All these questions are worth further investigating.

## Additional Information

**How to cite this article**: Liang, J. *et al*. A universal indicator of critical state transitions in noisy complex networked systems. *Sci. Rep.* **7**, 42857; doi: 10.1038/srep42857 (2017).

**Publisher's note:** Springer Nature remains neutral with regard to jurisdictional claims in published maps and institutional affiliations.

## Supplementary Material

Supporting Information
