# Supplementary material for: A universal indicator of critical state transitions in noisy complex networked systems
Source: Sci Rep. 2017 Feb 23;7:42857. doi: 10.1038/srep42857 (PMC5322368; doi:10.1038/srep42857)
Supplement: Supporting Information [file srep42857-s1.pdf]

Supplementary Information: A universal indicator of critical state transitions in noisy complex networked systems

Junhao Liang, Yanqing Hu, Guanrong Chen & Tianshou Zhou

Contents

A Theory of critical transition 2
A.1 One-dimensional case 2
A.2 Higher-dimensional case 7
B Dynamics of a simple system 12
C Numerical simulation methods 15
C.1 Network structure setting 15
C.2 Simulating the effect of random failures 17
D Extended validation 18
D.1 Simulations of artificial networks 18
D.2 Effect of noise on the characteristic returning time 20
References 22

A Theory of critical transition

In this section, we introduce the mathematical approach to analyzing the critical transition[1-3], focusing on the critical slowing down phenomenon[1,2,4-8] in a noisy setting. The analytical methods are based on the theory on stochastic differential equations and some known results from complex networks.

A.1 One-dimensional case

First, consider the following one-dimensional (1-D) nonlinear dynamical system subjected to small noise:

$$\frac{dx}{dt} = F(x,\alpha) + \sigma\xi(t) \tag{1}$$

where α is a tunable real parameter, which captures the changing environmental conditions; $\xi(t)$ is a standard Gaussian white noise with zero mean, satisfying $\langle \xi(t)\xi(t') \rangle = \delta(t-t')$, representing a perturbation; σ represents the noise intensity and function $F(x,\alpha)$ represents the system dynamics.

In the absence of noise, i.e., $\sigma = 0$, assume that the system is at one of the stable fixed points, x_s, given by

$$\begin{cases} F(x_s,\alpha) = 0 \\ \lambda_0 = \dfrac{\partial}{\partial x} F(x,\alpha)\Big|_{x=x_s} < 0 \end{cases} \tag{2}$$

The solution of this equation provides a resilience function, $x(\alpha)$, which represents all possible states of the unperturbed system as a function of α. The shape of this function is uniquely determined by the functional form of $F(x,\alpha)$. In contrast, the momentary state of the system is determined by the tunable parameter α. At some critical point $\alpha_{critical}$, the resilience function may feature a bifurcation (α_1 of Fig. S1) or become non-analytic (α_2 of Fig. S1), indicating[2,9] that the system loses its resilience via undergoing a sudden transition to a different, often undesirable, fixed

point of system (1).

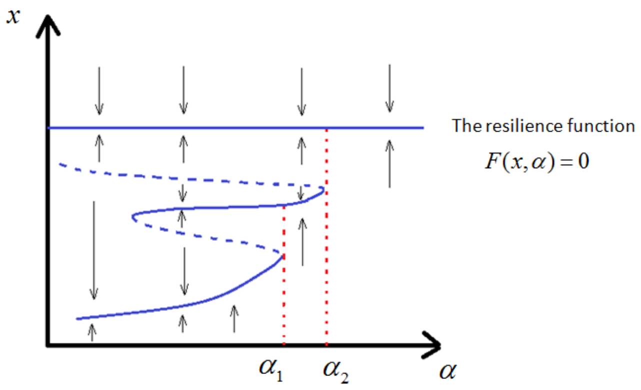

Figure S1. Possible shapes for the resilience function of the 1-D system (1), where the solid lines represent stable states whereas the dash lines represent unstable ones.

In the presence of noise, i.e., $\sigma \neq 0$, the Fokker-Planck equation corresponding to Eq. (1) is given by

$$\frac{\partial P(x,t)}{\partial t} = -\frac{\partial (FP)}{\partial x} + \frac{\sigma^2}{2}\frac{\partial^2 P}{\partial x^2} \tag{3}$$

where $P = P(x,t)$ is the probability distribution that the system is at state x at time t. Recall that by letting Ω be the size of a chemical reaction system, the intensity of the noise due to thermal movement of the species molecules is proportional to $1/\sqrt{\Omega}$ (called the noise factor) [10], giving $\sigma^2 \approx 1/\Omega$. Thus, similarly to the case of the linear noise approximation[10], if the system size Ω is sufficiently large or if the intensity σ^2 is sufficiently small (which will always be assumed in the following analysis), then one may set $x(t) = \varphi(t) + (1/\sqrt{\Omega})\mu(t)$, where $\varphi(t)$ is the deterministic part whereas $\mu(t)$ is the stochastic fluctuation part satisfying $\langle \mu(t) \rangle = 0$. To calculate the autocorrelation of the stochastic variable $\mu(t)$, denote by $Q(\mu,t)$ its probability density function. Then, the linear noise approximation yields the following Fokker-Planck equation:

$$\frac{\partial Q(\mu,t)}{\partial t} = -\lambda_0 \frac{\partial (\mu Q)}{\partial \mu} + \frac{\sigma^2}{2}\frac{\partial^2 Q}{\partial \mu^2} \tag{4}$$

At steady state, the autocorrelation is given by

$$\langle \mu^2(t) \rangle_{ss} = \frac{\sigma^2}{-2\lambda_0} \tag{4a}$$

and the lag-t autocorrelation by

$$\left\langle \mu(0)\mu(t)\right\rangle_{ss} = \frac{\sigma^2}{-2\lambda_0} e^{\lambda_0 t} \tag{4b}$$

Next, to show the perturbation effect of the noise, adopt the effective stability approximation procedure[11] and set

$$x(t) = x_s + x_p(t) + \sigma\mu(t) = (x_s + \sigma\mu) + x_p \tag{5}$$

where $x_p(t)$ represents a deterministic deviation from the steady state x_s. Note that, in the case where the perturbation is very small, the evolution of $x_p(t)$ is governed by the following linear equation with a stochastic coefficient:

$$\frac{dx_p(t)}{dt} = \lambda x_p(t) \tag{6}$$

where $\lambda = \partial F/\partial x\big|_{x=x_s+\sigma\mu(t)}$. Considering λ as a function of σ, making the Taylor expansion, and noting the smallness of σ, one obtains an approximation

$$\lambda(\sigma) \approx \lambda(0) + \sigma\lambda'(0) \tag{7}$$

where $\lambda(0) = \lambda_0 = \partial F/\partial x\big|_{x=x_s} =: F_x'(x_s, k_0)$, $\lambda'(0) = \mu(t)\partial^2 F/\partial x^2\big|_{x=x_s} = \mu(t) F_x''(x_s, k_0)$ (denoted by $\lambda_1(t)$ for convenience). With such an approximation, Eq. (6) can be rewritten as

$$\frac{dx_p(t)}{dt} = \left[\lambda_0 + \sigma\lambda_1(t)\right] x_p(t) \tag{8}$$

where $\lambda_1(t)$ depends on the steady-state correlation $\left\langle \mu(0)\mu(t)\right\rangle_{ss}$ given by Eq. (4b). Note that the stability of the zero solution to Eq. (8) is equivalent to that of x_s in the average sense. However, we are interested in the average stability. In this case, the equation for the average of $x_p(t)$ is governed by

$$\frac{d\langle x_p(t)\rangle}{dt} = \lambda_0 \left\langle x_p(t)\right\rangle + \sigma\left\langle \lambda_1(t) x_p(t)\right\rangle \tag{9}$$

To study this equation, one may assume various forms of $\lambda_1(t)$. In the case that $|\lambda_0| \gg |\lambda_1(t)|$, we adopt the Bourret approximation[12] for Eq. (9), obtaining

$$\frac{d\left\langle x_p(t)\right\rangle}{dt} \approx \lambda_0 \left\langle x_p(t)\right\rangle + \sigma^2 \int_0^t J_c(t-\tau)\left\langle x_p(\tau)\right\rangle d\tau \tag{10}$$

where $J_c(t-\tau) = \left\langle \lambda_1(t)\exp\left(\lambda_0(t-\tau)\right)\lambda_1(\tau)\right\rangle$ is the correlation function for fluctuations. At steady state, using Eq. (4b), one obtains

$$J_c(t-\tau) = \frac{\left[\sigma F_x''(x_s,k_0)\right]^2}{-2\lambda_0} e^{2\lambda_0(t-\tau)} \tag{11}$$

Substituting Eq. (11) into Eq. (10) yields

$$\frac{d\left\langle x_p(t)\right\rangle}{dt} = \lambda_0 \left\langle x_p(t)\right\rangle + a\int_0^t e^{2\lambda_0(t-\tau)} \left\langle x_p(\tau)\right\rangle d\tau \tag{12}$$

where $a = \left[\sigma^2 F_x''(x_s,k_0)\right]^2 \big/ (-2\lambda_0) > 0$.

Next, use the Laplace transformation method to solve Eq. (12). For this purpose, by setting $\widehat{x}_p(s) = \int_0^\infty x_p(t)e^{-st}dt$, one has

$$\langle \widehat{x}_p(s)\rangle = \left(s - \lambda_0 + \frac{a}{2\lambda_0 - s}\right)^{-1} \left\langle x_p(0)\right\rangle \tag{13}$$

The averaged perturbation mode $\langle x_p(t)\rangle$ is asymptotically stale if and only if the roots of the equation $s - \lambda_0 + \frac{a}{2\lambda_0 - s} = 0$ all have negative real parts. Hence, one gets the effective eigenvalue $\tilde{\lambda}_0$ expressed as

$$\tilde{\lambda}_0 = \frac{3\lambda_0 + \sqrt{\lambda_0^2 + 4a}}{2} = \frac{3\lambda_0 + \sqrt{\lambda_0^2 + 2\left[\sigma^2 F_x''(x_s,k_0)\right]^2 \big/ (-\lambda_0)}}{2} \tag{14}$$

The fact of $\lambda_0 + \sqrt{\lambda_0^2 + 4a} > 0$ implies that the noise positively contributes to the effective characteristic value of the stochastic system. In particular, for a small negative value of λ_0, although the deterministic system is stable, the stochastic system may approach and even cross the critical state if the noise is large. In order to better characterize such a critical transition, define

$$T = -1\big/\tilde{\lambda}_0 \tag{15}$$

This index actually represents the characteristic returning time of the 1-D system because

$\left\langle x_p(t)\right\rangle \sim \exp\left(\tilde{\lambda}_0 t\right) = \exp\left(-t/T\right)$. We will show that in contrast to $\tilde{\lambda}_0$, T is a better indicator to predict when the system goes to a critical state.

First, critical transition implies that a dynamic system has two potential stable states[1-3], which usually originates from the so-called fold-type bifurcation. According to the bifurcation theory for 1-D deterministic systems[13,14], when a system tends to go through a fold catastrophic bifurcation at x_s, the absolute value of the eigenvalue, $|\lambda_0|$, decreases to zero (namely, λ_0 increases to zero since it is negative), which corresponds to the increasing of both the lag-τ autocorrelation $K(\tau)$ defined by $K(\tau) = \left\langle\left\langle x(0)\, x(\tau)\right\rangle\right\rangle_{ss}$ and the variance Var defined by $\left\langle\left\langle x^2(t)\right\rangle\right\rangle_{ss}$ evaluated at steady state, since both are monotonic increasing functions of λ_0 as can be seen from Eq. (4a) and Eq. (4b). This is the same as the early-warning signals of critical slowing down: slower recovery rate from perturbations[4,5], higher autocorrelation[6,7] and higher variance[8].

Second, one can see from Eq. (14) that noise always has a positive effect on the characteristic value $\tilde{\lambda}_0$. When the system moves close to the critical state, $\tilde{\lambda}_0$ will approach zero from the negative direction. In particular, large noise can make $\tilde{\lambda}_0$ become positive and, in this case, T is negative. A negative characteristic returning time means that the system's recovery to the stable state is impossible.

Finally, notice that an approximate solution to Eq. (8) is given by $x_p(t) \approx x_p(0)e^{\tilde{\lambda}_0 t}$. From this expression, one can see that, for an initial deviation from the equilibrium, $d_0 = x(0) - x_s$ and a fixed $\tilde{\lambda}_0 < 0$, the time-evolutional deviation $d_t = x(t) - x_s$ will always return to zero exponentially after the time becomes sufficiently large. From the viewpoint of dynamical systems, the dominant eigenvalue (i.e., $\tilde{\lambda}_0$ in our case) characterizes the rate/speed of the recovery to the stable equilibrium. In applications, $\tilde{\lambda}_0$ can actually be considered as a recovery speed, which can be estimated from empirical data through simple linear regression (in this case, $\tilde{\lambda}_0$ is commonly called the Lyapunov exponent). Typically, one takes $x_p(t) \approx 0$ when $t > -1/\tilde{\lambda}_0$. Thus, the quantity $T = -1/\tilde{\lambda}_0$ characterizes the time returning to the equilibrium. In summary, compared

with the recovery rate $\tilde{\lambda}_0$, the index T serves as a better indicator for forecasting critical transitions in a highly nonlinear dynamical system subjected to noise.

A.2 Higher-dimensional case

Previous studies[2,3,15,16] showed that, when external environments change (captured by parameters), heterogeneous complex networks (nodes being non-similar) with high modularity tend to shift gradually, while homogeneous complex networks (nodes being similar) with high connectivity tend to occur at critical transitions up to some critical thresholds. This is because the homogeneity of nodes in a network affects the way in which the system with local alternative states responds to changing conditions[2]. Local damages of the homogeneous network tend to be repaired by subsidiary inputs from connected units until the system collapses at a critical stress level. In contrast, heterogeneous networks where components differ but are modular tend to have an adaptive capacity, so they can gradually adjust themselves to changes.

Here, we consider the case of homogeneous networks subjected to internal and external fluctuations (or noise). Similarly to the deterministic case[17], we first derive a one-dimensional (1-D) indicative equation from a higher-dimensional system, and then use the above developed theory to analyze various critical transitions in complex networked systems.

We begin by describing the complex networks in interest. A complex network can be described as a simple graph with N nodes, where there is at most one directed edge with positive weight from node i to node j for any i and j, and a self-edge from a node i to itself is permissible. Thus, this network can be characterized by an adjacency matrix $A=(a_{ij})_{N\times N}$, where element a_{ij} represents the weight of the directed edge from node j to node i. If there is no directed edge from node j to node i, then $a_{ij}=0$. If $a_{ij}>0$, j is a neighbor of i, meaning that i can be influenced by j. Since an undirected edge between i and j is equivalent in effect to both a directed edge from i to j and vice versa, the above adjacency matrix A is also suitable for the description of an undirected graph and in this case it is symmetric.

Define the in-degree and the out-degree of node i by $k_i^{in} = \sum_{j=1}^{N} a_{ij}$ and $k_i^{out} = \sum_{k=1}^{N} a_{ki}$, respectively. It is easy to show the relation of $\langle k_i^{in} \rangle = \langle k_i^{out} \rangle$, where the symbol $\langle \cdot \rangle$ represents the mean (i.e., overage) over all the network nodes (i.e., the sum over index i).

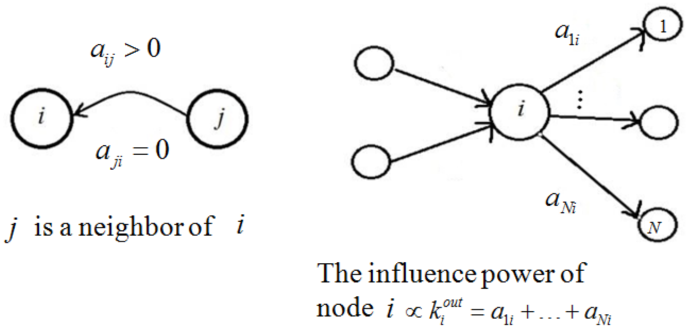

Figure S2. Graph representations of networks.

Suppose that the homogeneity of a network is so high that the dynamics of every node in the network can be approximately described by the following equation in the unified form:

$$\frac{dx_i}{dt} = f(x_i) + \sum_{j=1}^{N} a_{ij} \left[g\left(x_i, x_j\right) + \eta_{ij}(t) \right] + \xi_i(t) \quad (16)$$

where $f(x_i)$ describes the primitive deterministic dynamics of node i and $\xi_i(t)$ is a Gaussian white noise from fluctuations in the external environment; $a_{ij} g(x_i, x_j)$ represents the effective impact of node j on node i, and $a_{ij}\eta_{ij}(t)$ represents the effective noise in this impacting process. In Eq. (16), x_i describing the dynamics of node i may represent the quantity of a species, the expressional level of a gene, and so on.

In most of the previously-studied dynamical models, functions f and g were assumed to have some simple forms. Here, we assume that the form of function f may be complex but is of high linearity, namely $0 \neq |f''| \ll 1$. Similarly, function $g(x, y)$ is assumed to be of high linearity in variable y and is less affected by variable x, namely $\left|\partial^2 g / \partial y^2\right| \ll 1$ and $\left|g(x, y) - g(\tilde{x}, y)\right| \ll 1$. These assumptions are reasonable for many interactive processes in real-world complex networks. In addition, we assume that the amplitude of noise is much smaller than the size of the deterministic part, so that the conditions in the "critical slowing down" theory[1,2], the linear noise approximation[10] and the effective stability approximation[11], can be satisfied.

Note that the average of $\{x_i\}$ reflects the global properties of the whole network. The normalized average $\sum_{i=1}^{N} x_i / N$ does not seem to be a good description because the influence power of each node is not the same in the network. In order to highlight the influential differences between different nodes, we endow a weight w_j to each node with the conservative condition $\sum_{i=1}^{N} w_i = 1$. In our case, we set $w_j = k_j^{out} / \sum_j k_j^{out}$. We will use the weighted average $\langle x_i \rangle_w$, namely $\langle x_i \rangle_w = \sum_j k_j^{out} x_j / \sum_j k_j^{out} x_j \equiv \langle k_j^{out} x_j \rangle / \langle k_j^{out} \rangle$, to describe the mean behavior. Since we have assumed that the network is of a high homogeneity, the weight w_j can be approximated as $a_{ij} / \sum_j a_{ij}$ for all i.

Under the above assumptions, similarly to the deterministic case[17], we can derive the following equation from Eq. (16):

$$\frac{dx_i}{dt} = f(x_i) + k_i^{in} g\left(x_i, \langle x_i \rangle_w\right) + \zeta_i(t) \tag{17}$$

where $\zeta_i(t) = \sum_{j=1}^{N} a_{ij} \eta_{ij}(t) + \xi_i(t)$ is integrated noise, depending on the properties of the original noisy sources.

We are interested in the time-evolutional equation for the average quantity $\langle x_i \rangle_w$. From the linearity of $\langle \cdot \rangle_w$ with respect to the argument, we have

$$\frac{d\langle x_i \rangle_w}{dt} = \left\langle \frac{dx_i}{dt} \right\rangle_w = \langle f(x_i) \rangle_w + \left\langle k_i^{in} g\left(x_i, \langle x_i \rangle_w\right) \right\rangle_w + \langle \zeta_i(t) \rangle_w \tag{18}$$

According to our hypothesis on the function f, we have the approximation

$$\langle f(x_i) \rangle_w \approx f\left(\langle x_i \rangle_w\right) \tag{19}$$

With our hypothesis on the function $g(x, y)$, we also have

$$\left\langle k_j^{in} g\left(x_j, \langle x_j \rangle_w\right) \right\rangle_w = \frac{\sum_i k_i^{out} k_i^{in} g\left(x_i, \langle x_i \rangle_w\right)}{\sum_i k_i^{out}} \approx \frac{\sum_i k_i^{out} k_i^{in} g\left(\langle x_i \rangle_w, \langle x_i \rangle_w\right)}{\sum_i k_i^{out}} = \langle k_i^{in} \rangle_w \, g\left(\langle x_i \rangle_w, \langle x_i \rangle_w\right) \tag{20}$$

Now, denote $x = \langle x_i \rangle_w$, $k_0 = \langle k_i^{in} \rangle_w$ and $\zeta(t) = \langle \zeta_i(t) \rangle_w$. Then, we arrive at the following 1-D system

$$\frac{dx}{dt} = F(x, k_0) + \zeta(t) \tag{21}$$

where $F(x,k_0) = f(x) + k_0 g(x,x)$, and $\zeta(t)$ is the effective noise, which is expressed as a linear combination of internal and external noisy sources in the original large-scale system. Equation (21) is completely similar to Eq. (1), which we have analyzed above, so it has a well-defined characteristic returning time T.

Next, we analyze how the topological properties of the network affect the characteristic returning time T. For this purpose, we derive another expression of the parameter k_0. In fact, denote by $\langle k \rangle$ the average weight of the network, namely $\langle k \rangle = \langle k^{in} \rangle = \langle k^{out} \rangle$. Then, we have the following factorization[17]

$$k_0 = \frac{\langle k^{out} k^{in} \rangle}{\langle k^{out} \rangle} = \langle k \rangle + PCC\left(k^{in}, k^{out}\right)\sqrt{FF_{in} \cdot FF_{out}} \tag{22}$$

where PCC is the Pearson Correlation Coefficient, $PCC\left(k^{in}, k^{out}\right) = \frac{\langle k^{in} k^{out} \rangle - \langle k^{in} \rangle \langle k^{out} \rangle}{\sigma_{in} \sigma_{out}}$; $FF_{in} = \frac{\sigma_{in}^2}{\langle k_{in} \rangle}$ and $FF_{out} = \frac{\sigma_{out}^2}{\langle k_{out} \rangle}$ are the Fano Factors of the average in-degree and out-degree, where $\sigma_{in}^2 = \sum_{i=1}^{N} (k_i^{in} - \langle k^{in} \rangle)^2 / N$ and $\sigma_{out}^2 = \sum_{i=1}^{N} (k_i^{out} - \langle k^{out} \rangle)^2 / N$. Note that the value of $\langle k \rangle$ reflects the density of the network and a dense/sparse network tends to have a large/small $\langle k \rangle$. In contrast, FF_{in} and FF_{out} reflect the otherness of nodes and a network with greater degree differences between nodes tend to have greater FF_{in} and FF_{out}. The coefficient $PCC(k^{in}, k^{out}) \in [-1,1]$ describes the correlation between in-degree and out-degree. If $PCC(k^{in}, k^{out})$ is positive or negative, then the underlying network is called symmetric or asymmetric. Therefore, the sign of the in-out degree correlation reflects the network symmetry.

From the factorization (22), one can see that k_0 is a monotonically increasing function of $\langle k \rangle$ and $PCC(k^{in}, k^{out})$. A positive or negative $PCC(k^{in}, k^{out})$ implies that k_0 is a monotonically increasing or decreasing function of FF_{in} and FF_{out}.

The last issue is to determine the relationship between T and k_0. Note that such a relationship can be in principle analyzed by using Eq. (21). Note also that for fold catastrophic bifurcation, there are two distinct situations of the dynamic curves for the 1-D system described by Eq. (21), referring to Fig. S3. The difference between these two situations is caused by the type of the interaction between the nodes. Since the edges between nodes represent the interactive effects, the topological characteristic parameter k_0 represents the strength of this effect. In case (A) of Fig. S3, a greater k_0 (representing a stronger interactive effect) leads to a larger x_s, implying that the interactive effect is enhanced (mutualistic). On the contrary, the interactive effect is repressed (competitive) in case (B) of Fig. S3. Also, note that the type of the interactive effect is determined by the function $g(x,y)$, that is, $\partial g(x,y)/\partial y > 0$ corresponds to the mutualistic effect whereas $\partial g(x,y)/\partial y < 0$ to the competitive effect.

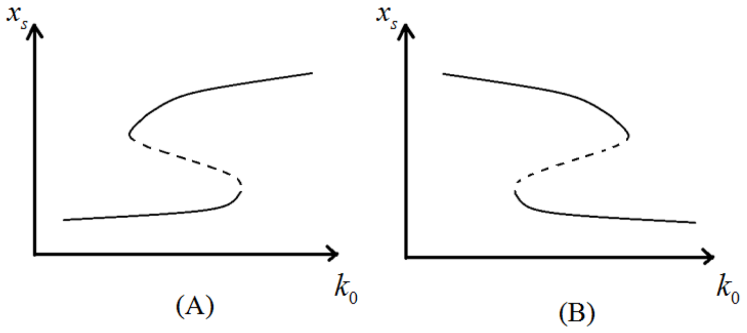

Figure S3. Two types of fold catastrophic bifurcation in system (21).

Before the critical transition occurs, the system has its stable state with a large value of x_s. In the mutualistic case, with the decrease of k_0, the system moves closer to the bifurcation point and λ_0 (thus $\tilde{\lambda}_0$) becomes greater (a universal law revealed by Ref. 13). Therefore, T is a monotonically decreasing function of k_0. Similarly, in the competitive case T is a monotonically increasing function of k_0.

From the above analysis, the relationship between the characteristic returning time T and the topological structure of the network can be summarized in Table S1.

Topological characteristic / Interaction type	Density	Difference	Topological characteristic / Symmetry
Mutualistic	-	-	Symmetric
		+	Asymmetric
Competitive	+	+	Symmetric
		-	Asymmetric

Table S1. Relationships between the characteristic returning time T and the topological structure of a network. Here, '+' /'-' represents increase/decrease of T. For example, in the mutualistic case, one can increase the network's density to decrease T (similarly in the competitive case). However, the influence of the difference on the characteristic returning time depends on the symmetry of the network. Note that these hold only before the critical state transition that corresponds to $T > 0$.

B Dynamics of a simple system

Now, consider Eq. (16) with $f(x) = b + x\left(1 - \frac{x}{K}\right)\left(\frac{x}{C} - 1\right)$. The reasons for choosing this form of f are as below. First, such a choice has realistic significance since f represents the Logistic growth with Allee effects in ecology[18,19], which describes the dynamic characteristics of many realistic processes. Second, this function is a cubic polynomial. For a general complicated function, one may only consider the first several terms in its Taylor expansion as approximation. Third, $|f''(x)| = \left|2\left(\frac{1}{C} + \frac{1}{K}\right) - \frac{6x}{CK}\right| \ll 1$ holds under some parametric conditions. In this case, the linearity of f is strong, which meets the requirement of the theory.

Reasons for the setting of $g(x, y) = \frac{\beta_1 x^n + \beta_2 y^n}{1 + \alpha_1 x^m + \alpha_2 y^m}$ are below. The function g of this form, also called a generalized Hill function, has been confirmed to be a good description for many interactive processes[20]. When the Hill coefficients n, m are small (we will set $n = 2, m = 1$ in the simulations below), $g(x, y)$ is of high linearity in variable y. If we choose the parameters to satisfy $\beta_1 \ll \beta_2$ and $\alpha_1 \ll \alpha_2$, then $g(x, y)$ is less affected by variable x. All these settings also meet the conditions of the theory.

As for the primitive noise at node i, the specific form of $\xi_i(t)$ is determined by stochastic

sources. Since there is no knowledge of these noise sources, they are often considered as rapidly fluctuating forces, and their effect is captured by Gaussian white noise. Specifically, it is assumed that $\xi_i(t)$ are mutually independent Gaussian white noise sources, satisfying

$$\left\langle \xi_i(t) \right\rangle = 0\,,\ \left\langle \xi_i(t)\xi_j(t') \right\rangle = \Gamma_i \delta_{ij} \delta\left(t - t'\right) \tag{23a}$$

where $\{\Gamma_i\}$ are constants characterizing the sizes of the fluctuations $\{\xi_i(t)\}$, δ_{ij} is the Kronecker delta, and $\delta(\cdot)$ is the common delta function. Similarly, assume that

$$\left\langle \eta_{ij}(t) \right\rangle = 0\,,\ \left\langle \eta_{ij}(t)\eta_{kl}(t') \right\rangle = \Gamma_{ij} \delta_{ik} \delta_{jl} \delta\left(t - t'\right) \tag{23b}$$

where $\{\Gamma_{ij}\}$ are constants characterizing the sizes of the fluctuations $\{\eta_{ij}(t)\}$. In addition, assume that these two kinds of noises are mutually independent, that is,

$$\left\langle \xi_l(t)\eta_{ij}(t') \right\rangle = 0 \tag{23c}$$

The integrated noise at node i, given by $\zeta_i(t) = \sum_{j=1}^{N} a_{ij}\eta_{ij}(t) + \xi_i(t)$, is a linear combination of some Gaussian white noise sources, so $\{\zeta_i(t)\}$ are Gaussian, satisfying

$$\left\langle \zeta_i(t) \right\rangle = 0\,,\ \left\langle \zeta_l(t)\zeta_i(t') \right\rangle = \left(\sum_j a_{ij}^2 \Gamma_{ij} + \Gamma_i\right) \delta_{li} \delta\left(t - t'\right) \tag{23d}$$

According to existing analytical results (see Section A in this Supplementary Information), the key to understand the global dynamics of the whole network is to study the 1-D equation

$$\frac{dx}{dt} = b + x\left(1 - \frac{x}{K}\right)\left(\frac{x}{C} - 1\right) + k_0 \frac{\beta x^n}{1 + \alpha x^m} + \zeta(t) \tag{24}$$

where $\beta = \beta_1 + \beta_2\,, \alpha = \alpha_1 + \alpha_2$; $k_0 = \langle k_j^{out} \rangle_w$ is a topological characteristic parameter, and $\zeta(t) = \left\langle \zeta_j(t) \right\rangle_w$ is the effective noise, which is Gaussian and satisfies

$$\langle \zeta(t) \rangle = 0\,,\ \left\langle \zeta(t)\zeta(t') \right\rangle = \sum_j \frac{\left(k_j^{out}\right)^2}{\left(\sum_l k_l^{out}\right)^2} \left(\sum_k a_{jk}^2 \Gamma_{jk} + \Gamma_j\right) \delta\left(t - t'\right) \tag{25}$$

Equation (24) is a quasilinear Langevin equation, and its deterministic dynamics is described as follows.

When $n = 2\,, m = 1$, denote by $F(x, k_0)$ the deterministic part of the right-hand side of Eq. (24). Then, $F(x, k_0) = 0$ is equivalent to

$$k_0(x) = -\frac{b}{\beta x^2} + \frac{1-b\alpha}{x} + \left(\frac{1}{CK} - \frac{\alpha}{C} - \frac{\alpha}{K}\right)x + \frac{\alpha x^2}{CK\beta} + \frac{1}{\beta}\left(\alpha - \frac{1}{C} - \frac{1}{K}\right) \qquad (26)$$

Differentiating both sides of Eq. (26) yields

$$k_0'(x) = \frac{2b}{\beta x^3} + \frac{b\alpha - 1}{\beta x^2} + \frac{2\alpha x}{CK\beta} + \frac{1}{\beta}\left(\frac{1}{CK} - \frac{\alpha}{C} - \frac{\alpha}{K}\right) \qquad (27)$$

From this, it follows that

$$\begin{aligned} &\lim_{x\to 0^+} k_0(x) = -\infty,\ \lim_{x\to +\infty} k_0(x) = +\infty \\ &\lim_{x\to 0^+} k_0'(x) = +\infty,\ \lim_{x\to +\infty} k_0'(x) = +\infty \end{aligned} \qquad (28)$$

Under some parametric conditions, there exists an interval $[A,B] \subset (0,+\infty)$ such that $k_0'(x)|_{[A,B]} < 0$. Analysis on the shape of the curve $F(x,k_0) = 0$ shows that, under certain parametric conditions, system (24) goes through a fold catastrophe bifurcation[1,14] (belonging to the mutualistic type). See Fig.1 in the main text for more details.

Note that the characteristic returning time T determined by Eq. (14) becomes

$$-\frac{1}{T} = \tilde{\lambda}_0 = \lambda_0 + \frac{\lambda_0 + \sqrt{{\lambda_0}^2 - 2(\Gamma \cdot F_x''(x_s,k_0))^2/\lambda_0}}{2} \qquad (29)$$

where

$$\lambda_0 = -1 + 2\left(\frac{1}{C} + \frac{1}{K}\right)x_s - \frac{3x_s^2}{CK} + \frac{k_0\beta x_s(2+\alpha x_s)}{(1+\alpha x_s)^2} \qquad (30a)$$

$$F_x''(x_s,k_0) = 2\left(\frac{1}{C} + \frac{1}{K}\right) - \frac{6x_s}{CK} + \frac{2k_0\beta}{(1+\alpha x_s)^3} \qquad (30b)$$

$$\Gamma = \sum_j \frac{\left(k_j^{out}\right)^2}{\left(\sum_l k_l^{out}\right)^2}\left(\sum_k a_{jk}^2 \Gamma_{jk} + \Gamma_j\right) \qquad (30c)$$

with x_s satisfying $F(x_s,k_0) = 0$.

C Numerical simulation methods

C.1 Network structure setting

In order to confirm the wide applicability of our new theory, we choose 9 typical and representative complex networks to implement numerical simulations. Among them, 3 networks are chosen from empirical data and the other 6 networks are artificially constructed, which include directed and undirected networks with different degree distributions and weight distributions. See Table S2 for more details.

Type		Network ID	Node Number N	Mean degree $\langle k \rangle$	Effective parameter k_0	Description
Empirical	Undirected	Net1	96	13.5	36.6	The data of Montane forest and grassland[23], where Net1 and Net2 are the mutualistic networks of the plants and pollinators respectively.
		Net2	276	6	18.2	
	Directed	Net3	1550	20.9	11.7	The gene regulatory networks of E. coli[21].
Artificial	Undirected	Net4	200	10		Random graph (E-R model) where all edges have the same weight.
		Net5	300	10		Random graph (E-R model) where all the weights of edges obey a power-law distribution.
		Net6	200	10		Scale-free networks where all edges have the same weight.
		Net7	300	12		Scale-free networks where all the weights of edges obey a power-law distribution.
	Directed	Net8	200	20		Directed scale-free networks without in-out degree correlations, where all edges have the same weight.
		Net9	300	20		Directed scale-free networks with negative in-out degree correlations, where all edges have the same weight.

Table S2. Complex network data used in numerical simulations. Net1~Net3 are empirical networks from experiments in ecology and molecular biology. The empirical data are download from Refs. 21,22, which were also used in the numerical simulation in Ref. 17. Net4~Net9 are representative random models extensively studied in the complex network theory.

For mutualistic networks Net1 and Net2, there is an initial bipartite $n \times m$ matrix M_{ik}, where the index i represents a plant and k represents a pollinator from the data of Montane forest and grassland[23]. By projecting the bipartite networks on their two node sets, the $n \times n$ plant network Net1 and the $m \times m$ pollinator network Net2 are constructed, where the nodes are connected by mutualistic interactions. The weight representing the mutualistic strength between nodes i and j in Net1 is determined by the following method: (i) the more mutual pollinators k that plants i and j share are, the stronger the mutualistic interaction between them is; (ii) the more plants pollinated by k are, the smaller its contribution to each plant is. For more detailed explanations, see Ref. 17. The weight construction for Net2 is similar to that for Net1, both in a symmetric way.

Net3 is the gene regulatory network in E. coli[21], a directed network where nodes represent the genes and edges represent the regulatory relations between them. The weights of edges are set initially to $a_{ij} = 10$ for all linked i, j pairs, just for simplicity.

The artificial networks are representative models studied in the complex network theory[24-26].

The Net4 (200 nodes) and Net5 (300 nodes) are random graphs (or the so-called Erdos-Renyi model) with mean degrees 5 and 10 (for initial nodes), respectively. The weights of edges in Net4 are all set to be 2 and the weights of edges in Net5 are chosen from a power-law distribution with mean 1 and power-law exponent 2.5.

The Net6 (200 nodes) and Net7 (300 nodes) are scale-free networks (the so-called Barabasi-Albert models) with mean degree 5 and 12 (for initial nodes), respectively, and their degree distributions are both power-law with exponent 2.5. The weights of edges in Net6 are all set to be 2 and the weights of edges in Net7 are chosen from a power-law distribution with mean 1 and power-law exponent 2.6.

The Net8 (200 nodes) and Net9 (300 nodes) are directed scale-free networks with mean (in and out) degrees 5 and 10 (for initial nodes), respectively, and their degree distributions are power laws with exponent 2.5, while Net9 has negative in-out degree correlations (nodes with large out-degree often have small in-degree) and Net8 is the one without in-out degree correlations. The weights of edges of them are set to be 4 and 2, respectively.

C.2 Simulating the effect of random failures

To simulate the effect of random failures or attacks, we adopt the following settings. For random failures, we delete nodes from the network with a fraction ρ randomly (to simulate the internal failures). For random attacks, we randomly reduce a fraction ρ of the weights of the initial network (to simulate the external environmental deteriorations), that is, we let $a_{ij} \to (1-r)a_{ij}$ for non-zero elements in the adjacency matrix A of the network, where r is chosen from a Beta distribution with mean ρ. It is known that the Beta distribution $Be(a,b)$ with parameters a,b has the mean $\frac{a}{a+b}$ and the variance $\frac{ab}{(a+b)^2(a+b+1)}$. Note that this distribution is unimodal for $a>1, b>1$. For a fixed ρ, one can choose two suitable parameters $a>1, b>1$ to construct the needed Beta distribution. Here, we set $\rho = \frac{a}{a+b}$ and the variance can be reduced by increasing a,b simultaneously. For an undirected network, the adjacency matrix is kept symmetric. After the failing, we use the given network data to perform simulation and calculation. When a fraction ρ of the network weights is randomly removed, the network changes its topology in a stochastic way, thus influencing its topological characteristic parameter k_0.

By using the given network data $\{a_{ij}\}$, the stochastic differential equations (i.e., Eq. (1) in the main text) can be solved by the Euler method[27,28]. In simulation, we set the system's parameters as

$$\begin{aligned} &b = 0.1, \quad C = 1, \quad K = 5, \quad n = 2, \quad m = 1, \\ &\alpha_1 = \beta_1 = 0.01, \quad \alpha_2 = \beta_2 = 0.19, \end{aligned} \tag{31}$$

and adjust Γ_{ij}, Γ_i to obtain a desired noise intensity.

When numerically solving Eq. (1) in the main text, we set two initial conditions: a low initial state value with all $x_i(0) = 0.01$ and a high initial state value with all $x_i(0) = 5$. This allows us to test whether the critical transition takes place. In fact, at the before-transition state, the system has its stationary position around a unique stable state with large magnitudes of components. The appearance of the stable state with a large magnitude implies that the critical transition has occurred.

To get a better representation of the changes in the system's state, we calculate the average

$\langle x\rangle = \sum_{i\in GC} x_i \big/ |GC|$, where GC is a giant component of the network. This quantity reflects the global resilience of the system and can describe whether critical transition occurs. See Fig. 2 in the main text for more details.

Finally, we calculate the average of the weighted means $\langle x\rangle_w$ of the network components and use the stationary state data (from which we can get the stationary average $E[\langle x\rangle_w]$) to test whether the theoretical prediction of the 1-D equation is effective. See Fig. 3a in the main text for more details.

D Extended validation

D.1 Simulations of artificial networks

In the main text we have shown that the higher-dimensional stochastic dynamic behavior of a complex networked system can be captured by a 1-dimensional stochastic equations, Eq. (21). The form of the 1-D equation is solely determined by the initial dynamics of the original large-scale system, namely $f(\cdot)$ and $g(\cdot,\cdot)$ in Eq. (16). The complex networked system has an important topological characteristic parameter, $k_0 = \left\langle k_j^{in} \right\rangle_w$, which can be calculated based on the network adjacency matrix $\{a_{ij}\}$. Note that this parameter preserves the topological structure properties of the complex network. Note also that different network topological structures give rise to different dynamic patterns.

To validate our prediction theory, we also conducted a set of numerical tests on artificial complex networks, which are of different scales, levels, degree differences and structural correlations. All these simulated complex networks are classified into 6 types. See Net4-9 in Section C.1 in this Supplementary Information for details.

For the 6 artificial networks, we performed numerical simulations with numerical results shown in Fig. S4, where the row diagrams from top to bottom correspond to the random graph with uniform weights, the random graph with power-law weights, the scale-free network with uniform weights, the scale-free network with power-law weights, the directed scale-free network with uniform weights and without in-out degree correlations, and the directed scale-free network with uniform

weights and with negative in-out degree correlations, respectively. The column diagrams from left to right correspond to low loss, high loss, high loss with large noise, universal dynamics, and the characteristic returning time of the complex networks, respectively. These numerical results also have well verified our theoretical predictions.

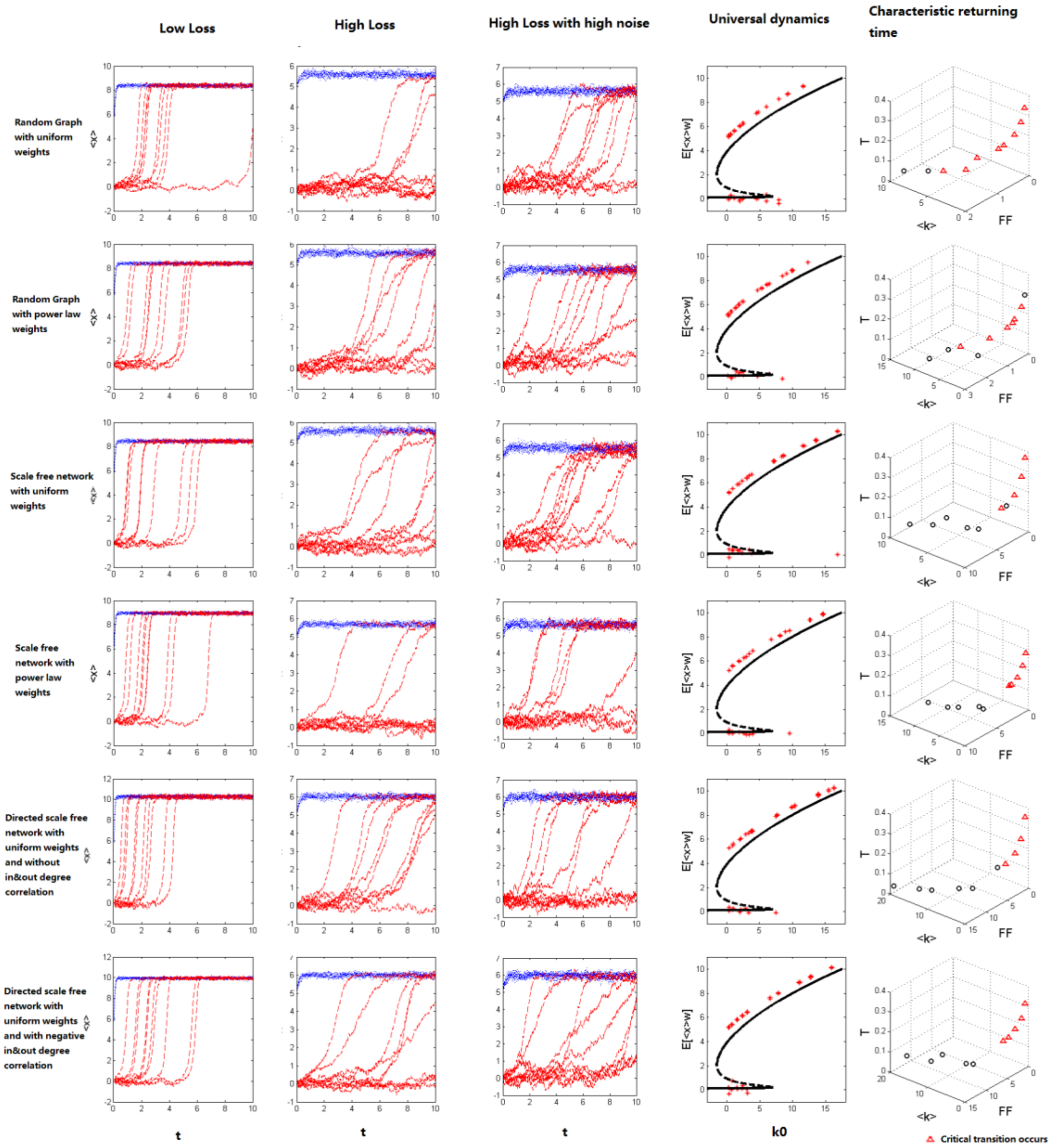

Figure S4. Critical transitions in artificial networks with different topological properties. The numerical results of these networks confirm the universality of the indicator that we introduced in the main text and in this Supporting Information. Here, low loss is set as 40% (corresponds to $\rho = 0.4$), high loss is set as 90% (corresponds to $\rho = 0.9$), high noise corresponds to the setting of $\Gamma_{ij} = 0.05, \Gamma_i = 0.1$, the noise in the other cases corresponds to the setting of $\Gamma_{ij} = 0.001, \Gamma_i = 0.01$. Other settings are the same as Fig. 2 and Fig.3 in the main text.

D.2 Effect of noise on the characteristic returning time

We furthermore compare the characteristic returning time in the case considering the noise effect, $T = -\tilde{\lambda}_0^{-1}$, and the case without considering the noise effect, $T = -\lambda_0^{-1}$. See section A in this Supplementary Information for more details. It is found that noise has an enhancing effect on or has a positive contribution to T, remarkably when the system is close to the critical transition state, referring to Fig. S5. In a word, the index T incorporating the noise effects is a good indicator for the critical transitions in the networks since noise can amplify the critical signals.

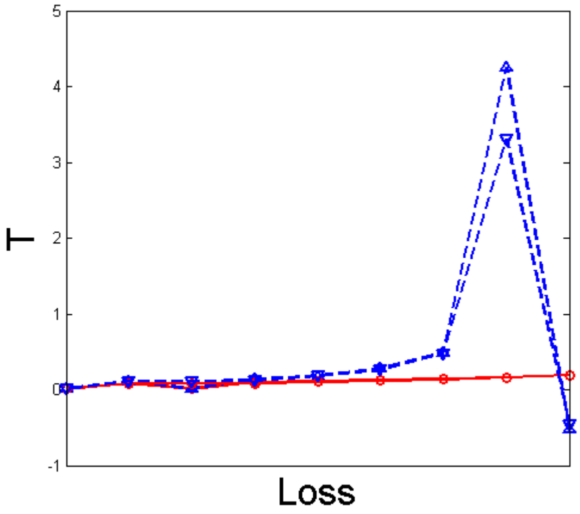

Figure S5. An example showing the effect of noise on the characteristic returning time T (which has been magnified by 10 times), where dashed(blue)/solid(red) curves represent the characteristic returning time in the cases with/without considering the noise effects. We enhance the loss fraction of the network Net1 and calculate the corresponding characteristic returning time. It can be seen that noise has a remarkable effect on T when the system is close to a critical state. The parametric settings are the same as Fig. 3 in the main text.

References

1. Scheffer, M., et al. Early-warning signals for critical transitions. *Nature* **461**, 53-59 (2009).
2. Scheffer, M., et al. Anticipating critical transitions. *Science* **338**, 344-348 (2012).
3. Scheffer, M. *Critical Transitions in Nature and Society* (Princeton Univ Press, Princeton, 2009).
4. van Nes E. H. & Scheffer, M. Slow recovery from perturbations as a generic indicator of a nearby catastrophic shift. *Am. Nat.* **169**, 738–747 (2007).
5. Annelies J., et al. Recovery rates reflect distance to a tipping point in a living system. *Nature* **481**, 357-359 (2012).

6. Dakos, V., et al. Slowing down as an early warning signal for abrupt climate change. *Proc. Nat. Acad. Sci. USA* **105**, 14308-14312 (2008).
7. Ives, A. R. Measuring resilience in stochastic systems. *Ecol. Monogr.* **65**, 217-233(1995).
8. Carpenter, S. R. & Brock, W. A. Rising variance: a leading indicator of ecological transition. *Ecol. Lett.* **9**, 311-318 (2006).
9. May, R. M., Levin, S. A. & Sugihara, G. Complex system: Ecology for bankers. *Nature* **451**, 893-895 (2008).
10. van Kampen, N. G. *Stochastic Processes in Physics and Chemistry (Third Edition)* (Elsevier, Singapore, 2009).
11. Scott, M., Hwa, T. & Ingalls, B. Deterministic characterization of stochastic genetic circuits. *Proc. Nat. Acad. Sci. USA* **104**, 7402-7407 (2007).
12. Poulin, F. J. & Scott, M. Stochastic parametric resonance in shear flows. *Nonlinear Processes in Geophys* **12**, 871-876 (2005).
13. Wissel, C. A universal law of the characteristic return time near thresholds. *Oecologia* **65**, 101–107 (1984).
14. Kuznetsov, Y. A. *Elements of Applied Bifurcation Theory* (Springer, New York, 1995).
15. van Nes, E. H. & Scheffer, M. Implications of spatial heterogeneity for regime shifts in ecosystems. *Ecology* **86**, 1797-1807 (2005).
16. Dunne, J. A., Williams, R. J. & Martinez, N. D. Network structure and biodiversity loss in food webs: robustness increases with connectance. *Ecol. Lett.* **5**, 558-567 (2002).
17. Gao, J., Barzel, B. & Barabási, A.-L. Universal resilience patterns in complex networks. *Nature* **530**, 307-312 (2016).
18. Hui, C. Carrying capacity, population equilibrium, and environment's maximal load. *Ecol. Model.* **192**(1), 317–320 (2006).
19. Allee, W. C., et al. *Principles of Animal Ecology* (Saundere, 1949).
20. Setty, Y., Mayo, A. E., Surrette, M. G. & Alon, U. Detailed map of a cis-regulatory input function. *Proc. Nat. Acad. Sci. USA* **100**, 7702-7707 (2003).
21. Gama-Castro, S., et al. (version 6.0): Gene regulation model of escherichia coli k-12 beyond transcription, active (experimental) annotated promoters and textpresso navigation. *Nucleic Acids Research* **36**, Databaseissue:D120–4 (2008).
22. Interaction web database. http://www.nceas.ucsb.edu/interactionweb/resources.html#plant_ant. Accessed: 2010-09-30.
23. Clements, F. E. & Long, F. L. *Experimental Pollination: An Outline of the Ecology of Flowers and Insects* (Carnegie Institution of Washington, 1923).
24. Albert, R. & Barabási, A.-L. Statistical mechanics of complex networks. *Rev. Mod. Phys.***74**, 47-97 (2002).
25. Cohen, R. & Havlin, S. *Complex Networks: Structure, Robustness and Function* (Cambridge Univ Press, New York, 2010).
26. Bollobas, B. *Random Graphs* (Academic Press, New York, 1985).
27. Kloeden, P. E. & Platen, E. *Numerical Solution of Stochastic Differential Equations* (Springer, New York, 1992).
28. Higham, D. J. An algorithmic introduction to numerical simulation of stochastic differential equations. *SIAM Review* **43**, 3:525-546 (2001).
